# Tissue-Resident Memory T Cells in Skin Diseases: A Systematic Review

**DOI:** 10.3390/ijms22169004

**Published:** 2021-08-20

**Authors:** Thomas Emmanuel, Josephine Mistegård, Anne Bregnhøj, Claus Johansen, Lars Iversen

**Affiliations:** Department of Dermatology, Aarhus University Hospital, DK-8200 Aarhus, Denmark; josjor@rm.dk (J.M.); annebreg@rm.dk (A.B.); claus.johansen@clin.au.dk (C.J.); lars.iversen@clin.au.dk (L.I.)

**Keywords:** tissue-resident memory T cells, TRMs, TRM, T cells, inflammation, skin disease, systematic review

## Abstract

In health, the non-recirculating nature and long-term persistence of tissue-resident memory T cells (TRMs) in tissues protects against invading pathogens. In disease, pathogenic TRMs contribute to the recurring traits of many skin diseases. We aimed to conduct a systematic literature review on the current understanding of the role of TRMs in skin diseases and identify gaps as well as future research paths. EMBASE, PubMed, SCOPUS, Web of Science, Clinicaltrials.gov and WHO Trials Registry were searched systematically for relevant studies from their inception to October 2020. Included studies were reviewed independently by two authors. This study was conducted in accordance with the PRISMA-S guidelines. This protocol was registered with the PROSPERO database (ref: CRD42020206416). We identified 96 studies meeting the inclusion criteria. TRMs have mostly been investigated in murine skin and in relation to infectious skin diseases. Pathogenic TRMs have been characterized in various skin diseases including psoriasis, vitiligo and cutaneous T-cell lymphoma. Studies are needed to discover biomarkers that may delineate TRMs poised for pathogenic activity in skin diseases and establish to which extent TRMs are contingent on the local skin microenvironment. Additionally, future studies may investigate the effects of current treatments on the persistence of pathogenic TRMs in human skin.

## 1. Introduction

Tissue-resident memory T cells (TRMs) are a subset of memory T cells. TRMs can be of either cluster of differentiation (CD)4^+^ or CD8^+^ lineage. CD4^+^ T cells assist in fostering the development of cytotoxic memory CD8^+^ T cells following infection. However, the role of CD4 and CD8 expression has not yet been fully elucidated [1]. TRMs have been implicated in several different tissues and associated diseases. In humans, TRMs have been found in the lungs, skin, salivary glands, brain, the female reproductive tract, intestines, bone marrow and liver [2,3,4,5,6,7,8,9]. TRMs provide immune protection against pathogens located in peripheral tissues, often more rapidly than other cell types.

### 1.1. Generation and Definition of TRMs

The differentiation of TRMs have so far not yet fully been elucidated, and the existence of a TRM precursor cell is debated. However, during antigen encounters, TRMs can differentiate from circulating killer cell lectin like receptor G1 (KLRG1)^−^ precursor cells, central memory T cells (TCMs), and effector memory T cells (TEMs) in peripheral tissues [10]. TEMs migrate to inflamed peripheral tissues and show immediate effector functions, whereas TCMs migrate to T cell areas of secondary lymphoid organs where they then differentiate into TEMs and exert effector functions [11,12,13]. In the local microenvironment, tissue-derived signals and cytokines such as transforming growth factor-beta 1 (TGF beta 1) can instruct the tissue residency program in precursor cells [14,15].

In contrast to circulating memory T cells, TRMs are defined by their apparent inability to recirculate to other tissues [16,17]. TRMs are heterogeneous and reliant on the microenvironment in different tissues and diseases. On renewed inflammation, pathogen-specific TRMs proliferate and exert effector functions [10,18]. In the skin, TRMs survey for pathogens by migrating within the constrained epidermal compartment and squeezing between keratinocytes using multiple dynamic dendritic projections. This ensures enhanced and swift protection against subsequent exposure to the same pathogen [19,20].

No defining criteria have yet been established to consistently identify TRMs across tissues, but the expression CD3, CD4 or CD8 in addition to the residency markers CD69 and CD103 are often utilized. While CD103 is variably expressed, CD69 has been revealed as a consistent marker for resident cells [21,22].

### 1.2. TRMs in Skin Diseases

The long-lived, non-recirculating characteristic of autoreactive dysregulated TRMs can also cause skin diseases [23]. Diseases such as psoriasis, vitiligo and cutaneous T-cell lymphoma (CTCL) are characterized by well-demarcated plaques [24], and lesions often recur in the same location once therapy is discontinued, indicating a type of disease memory [25]. These lesions are often highly enriched with pathogenic TRMs [26,27], and it has been suggested that TRMs harbor a disease memory [27]. The skin allows easy access to various treatments. Consequently, potential modulatory treatment options targeting TRMs are of high interest, as such treatment may hypothetically affect the long-term outcome of even chronic skin diseases. The present systematic review highlights current knowledge on the role of TRMs in skin diseases and discusses gaps and future paths for TRM-targeted treatment.

We performed a systematic review of the role of tissue-resident memory T cells in skin diseases and found pathogenic TRMs to have a role in several skin diseases including skin infections, psoriasis, melanoma and CTCL. More studies are needed to discover biomarkers that may better delineate TRMs poised for pathogenic activity in skin diseases.

## 2. Materials and Methods

### 2.1. Protocol and Registration:

This protocol was registered with the PROSPERO database (ref: CRD42020206416). A search was conducted on PROSPERO to certify that similar systematic review study protocols had not been registered. The PRISMA-S was used to guide the review process.

### 2.2. Search Strategy

A systematic literature search was conducted in EMBASE, PubMed, SCOPUS and Web of Science. A search on trial registers http://www.ClinicalTrials.gov and http://www.who.int/trialsearch/ was conducted 25 October 2020 to identify unpublished or ongoing trials. Searches covered the period from register inception to October 2020 using the search string provided in Table S1. A university librarian was consulted to support the development of the search strategy. Reference lists and citations in included papers were then hand-searched and a few relevant reviews were searched for additional relevant papers. Search results were imported into Covidence (https://www.covidence.org/, accessed on 25 October 2020). Duplicates were removed in Mendeley at both the first and last step, as using Covidence and Mendeley alone was unsuccessful in fully de-duplicating results after importation.

### 2.3. Study Inclusion Criteria

This review includes peer-reviewed studies in the English language only. Furthermore, the included papers describes human, animal or in vitro studies of relevance to human skin diseases.

### 2.4. Study Exclusion Criteria

This study excluded opinion papers, discussion papers, review articles, conference abstracts, perspective articles, books or grey literature and editorial comments. Additionally, studies with no apparent relevance to skin diseases or no mention of TRMs in skin were excluded. Finally, if no full-text digital article could be acquired, the study was excluded.

### 2.5. Study Selection

Study selection included four phases. First, abstracts were screened by JM and TE independently and matched against the inclusion criteria. All studies that met the inclusion criteria were progressed for full-text review, and the exclusion criteria were applied independently by JM and TE. Lastly, duplicates were removed. Any new articles were processed as outlined above. Any difference of judgement between JM and TE was resolved by consensual agreement. Because of the expected heterogeneity of the initial studies selected, no specific tools for quality assessment were introduced. We excluded articles solely focusing on TRM biology or novel methods without any direct experiment related to a specific skin disease or model of skin disease. We focused on articles where the authors used the term TRM or a comparable term to describe the investigated cells. All papers from inception to the initiation of this review were included.

## 3. Results

**Figure 1:**
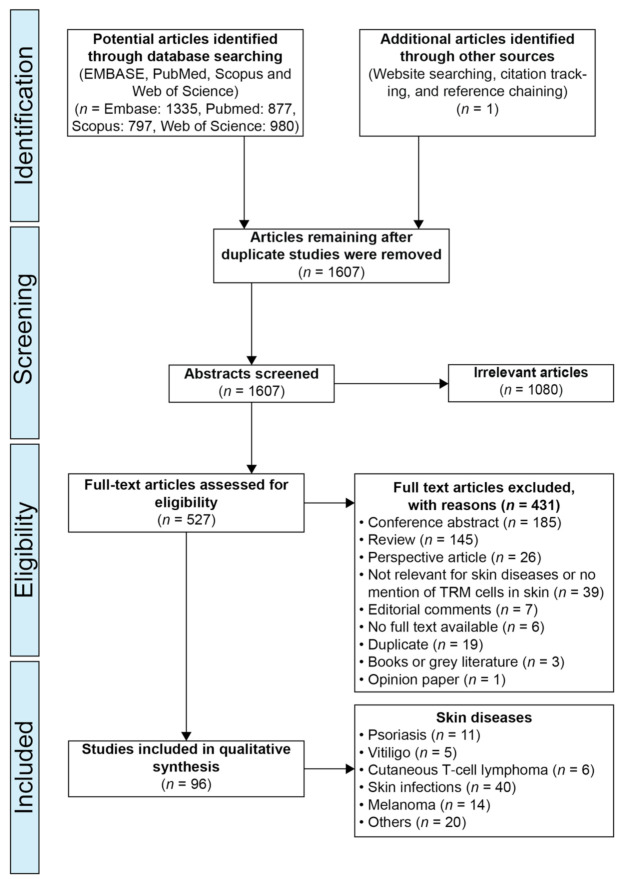
Literature search strategy. A total of 1335 records were found in Embase, 877 in Pubmed, 797 in Scopus and 980 in Web of Science. In total 1607 records were found. Abstracts were screened for eligibility using inclusion and exclusion criteria (see Material and Methods). The full texts of studies included at this stage were then reviewed, and 431 of the reviewed texts were excluded for being a conference abstract (185), a review (145), a perspective article (26), irrelevant for skin diseases or failing to mention TRMs in the skin (39), an editorial comment (7), having no full text available (6), a duplicate (19), books or grey literature (3) and an opinion paper (1). In total, 95 publications from the literature search were included. One additional article was included after screening the references of studies from the search result; and following external review, this produced to a total of 96 articles. TRMs = Tissue-Resident Memory T cells.

The results of the search strategy are summarized in Figure 1. No ongoing clinical studies were identified in the clinical research databases using any of the search strings. Table 1 shows a list of markers used to distinguish TRMs, and Table 2 shows the articles listed by disease. Table S1 shows a list of descriptive data extracted from the included papers. Below is a narrative summary of results from the articles listed by theme.

### 3.1. TRMs and Skin Infections

Studies have now established that skin infections—viral, bacterial, fungal and parasitic—generate TRM populations that are effective in preventing both local and distant skin reinfections (Table 2).

The mouse model of herpes simplex virus (HSV) is one of the most frequently used models to study TRMs in skin infection. It has been used to show that HSV infection generates a population of TRMs that engaged virus-infected cells, mediated local protection and remained confined to the epidermal and dermal niches [18,65,71,78,79,89,93,97,103,107,110]. CD4^+^ TRMs were found to recirculate in the dermis, whereas CD8^+^ TRMs remained mostly confined in the epidermis in the area of previous HSV infection [65]. Skin TRMs were maintained as a stable population after recall and remained highly functional at recall [97,110]. In HSV, CD49a was found to support CD8^+^ TRM persistence within skin, to regulate dendritic extensions on epidermal CD8^+^ TRMs and to increase the frequency of interferon-γ (IFN-γ)^+^ CD8^+^ TRMs [80]. CD69 prolonged T cell retention and local memory formation [93]. Furthermore, CD69 deficiency led to a reduction in the generation of CD103^+^ TRMs in the skin [81]. In human skin, TRMs are possibly involved in Wolf’s isotopic response following herpes infection [74].

Other mouse models of skin infection—such as vaccinia virus vaccination and skin scarification—indicate that skin infection results in the generation of long-lived skin TRMs [14,38,46,75,76,77,82,83,84,85]. In contrast, a general inflammatory condition like sepsis has minimal impact on the number and function of CD8^+^ TRMs in the skin [86]. Instead, the differentiation state and persistence of these long-lived TRMs depend on several molecules and antigens in the microenvironment, such as an increased amount of TGF-β produced by keratinocytes and dendritic cells [47,48,83,87]. In addition, cross-priming by dendritic cells is important for adequate skin TRM generation [88].

Deficiency of dedicator of cytokinesis 8 (DOCK8), a protein involved in regulating the cell actin skeleton, was found to decrease the numbers of CD69^+^ and CD103^+^ TRMs surviving in the skin after skin infection. This may be due to a change in cell shape integrity, which is important for epidermal lymphocyte migration [90]. In contrast, interleukin (IL)-15 has been found to recruit TRMs to inflamed tissues, paving the way for both vaccination and immunotherapeutic modulation of skin TRMs [91]. Also, targeting the metabolism through fatty acid-binding protein 4 and 5, which are important for persistence of the TRMs in skin, may serve as a future potential way of modulating the number of TRMs in the skin [92].

Cutaneous poxvirus infection and response on TRMs have also been studied, and it was shown that CD8^+^ TRMs can provide protection against the virus [53].

Three studies explored infection with the commensal fungus *Candida albicans* and showed that *Candida albicans* can generate long-lived protective CD4^+^ TRMs in the skin [112]; in particular, the CD4^+^ CD69^+^ TRM-like T cells showed increased effector function in response to *Candida albicans* [54]. This ability of TRMs to produce effector cytokines in response to *Candida albicans* was also supported in a study by Senechal et al. [49].

Three studies have explored TRMs in relation to parasitic infection. One study showed that tick infection can generate IL-3^+^ CD4^+^ TRMs distant from the primary tick infection site, potentially leading to efficient control at re-infection [50]. CD4^+^ TRMs present in the skin after resolution of *Leishmania Major* infection are retained long after disease resolution, where they produce IFN-γ and enhance the recruitment of circulating memory cells to the site of Leishmania challenge [55]. Furthermore, leishmania-specific TRMs can rapidly recruit and activate inflammatory monocytes at the site of infection [56].

Only one study investigated the role of TRMs in bacterial infection and here, TRMs were induced in a *Staphylococcus aureus* infection site. However, local TRMs failed to provide immunological memory against a secondary local infection [113].

Taken together, most studies on skin infection have been conducted using various mouse models of skin infection, with the HSV infection model being used most frequently. It remains unclear why some infections lead to long-lasting local immunological memory with TRMs, whereas infection with other pathogens does not.

### 3.2. Psoriasis

The psoriasis pathogenesis depends on environmental and genetic factors and involves communication between different immune cells through cytokines such as tumor necrosis factor-α (TNFα), IFN-γ, IL-17, IL-22 and IL-23. This results in a self-sustaining inflammation cycle [121]. Psoriasis lesions are known to recur in previously affected areas following relapse after treatment cessation, pointing to a molecular scar with disease memory in clinically healed skin [25]. In the Aldara-induced psoriasis-like skin inflammation mouse model, Vγ4^+^ Vδ4^+^ cytokine—producing T cells have the potential to mount a memory response [117]. During active psoriasis, an increase in epidermal CD8^+^ T cells expressing TRM markers in the epidermis has been shown [57]. Furthermore, CD8^+^ T cells producing IL-17 and CD4^+^ T cells producing IL-22 remain in resolved lesions and can be stimulated to produce psoriasis-related cytokines even after treatment with biologics such as infliximab for several years [51]. Interestingly, the effect on the presence of TRMs of treatment with different biological drugs has also been studied and, so far, no difference in the number of CD103^+^ cells in residual psoriatic plaques has been shown [116]. The presence of IL-17A-producing CD8^+^ CD103^+^ TRMs in the epidermis has been found to contribute to the prognosis of psoriasis [52]. However, in previously unaffected non-lesional psoriasis skin, the IL-17A-generating potential of CD8^+^ CD103^+^ cells increases with disease duration [58]. Furthermore, a case study found that the percentage of CD8^+^ CD103^+^ TRMs was higher in psoriatic epidermis than in an isomorphic response of Köbner [94], which begs the question if psoriasis reoccurs only in TRM-seeded areas or if psoriasis simply results in an increase of unrelated TRMs in the skin.

Several avenues for targeting the TRM cells in psoriasis have been proposed such as manipulating the lipid metabolism through fatty-acid-binding proteins 4 and 5. These molecules mediate lipid uptake and intracellular transport. Deficiency of these molecules decreases the long-term survival of CD8^+^ TRM cells in human psoriatic skin [114]. Additionally, dihydroartemisinin but not methotrexate reduced the presence of CD8^+^ CLA+, CD8^+^ CD69^+^ and CD8^+^ CD103^+^ TRM cells in mouse skin and reduced human CD8^+^ CD103^+^ TRM cells in humanized mice skin [59], indicating that novel treatments might affect the TRMs and possibly modify the course of psoriasis.

Taken together, these studies suggest that active lesional, resolved lesional and non-lesional psoriasis skin harbor a population of pathogenic TRM cells that may have the potential to become activated after recognizing various antigens, thereby resulting in the characteristic well-demarcated disease recurrence after therapy cessation. Whether early intensive therapy can change or modulate the pathogenic TRMs remains an interesting question that is currently being investigated [118].

### 3.3. Vitiligo

Vitiligo is an autoimmune skin disease caused by destruction of pigment-producing melanocytes in the epidermis, leading to hypopigmented areas of the skin. Vitiligo is often a chronic disease that requires lifelong therapy; approximately 40% of patients with vitiligo relapse within one year after treatment cessation [122,123]. Hypopigmented lesions typically recur at the same location after discontinued therapy, which may indicate autoimmune memory.

Vitiligo lesions harbor a population of autoreactive TRMs [120]. Studies of human TCR Vβ usage have found that autoreactive TRMs within vitiligo lesions are polyclonal as T-cell clones vary across patients and even across lesions within the same patient [27,28]. TRMs with cytotoxic potential in vitiligo express CD49a. In addition to cellular cytotoxicity, TRMs secrete IFN-γ, perforin and granzyme B, which are essential for inducing melanocyte apoptosis in vitiligo. Formation of TRMs and secretion of IFN-γ are promoted by IL-15 [27].

A recent study investigated the functional relationship of TRMs and recirculating memory T-cells (TCMs), and findings suggest that TRMs cooperate with TCM populations in maintaining depigmentation in vitiligo [28]. The study found that inhibition of T-cell recruitment to the skin with the sphingosine-1-phosphate receptor (S1P1)-inhibitor FTY720 and TCM depletion with low-dose Thy1.1 antibody reversed the disease and led to repigmentation. TRMs produce C-X-C motif chemokine ligand (CXCL) 9 and CXCL10, which bind to the C-X-C chemokine receptor 3 expressed on TCMs. TRMs may serve a sensing function and recruit TCMs through chemokine signaling. Furthermore, S1P1 modulators, which regulate migration of lymphocytes from lymph nodes, may serve as potential treatments for vitiligo [28,95]. IL-15 has also been proposed as a potential therapeutic target. Melanocyte-specific TRMs express high levels of the CD122 chain of the IL-15-receptor. Hence, targeting IL-15 signaling with an anti-CD122 antibody has been suggested as a treatment strategy for vitiligo. It has been reported that short-term treatment with anti-CD122 in a mouse model of vitiligo inhibits the production of IFN-γ, and long-term treatment depletes TRMs from skin lesions [95]. In contrast, a recent study in mice found that TRM populations remained in the skin, even after significant repigmentation was observed during Tofacitinib and Ruxolitinib treatment [29].

Taken together, a growing body of evidence indicates a role of TRM in the pathogenesis and maintenance of vitiligo.

### 3.4. Melanoma

Immune-cell access to the epidermal compartment is critical for effective anticancer protection. TRMs are present in the epidermal layer and it seems intuitive to examine the role of TRMs in melanoma.

TRMs recognizing tumor-specific antigens have been shown to suppress melanoma growth [30,96] and provide potentially body-wide protection against melanoma [30,60,98], as well as local protection in the proximity of the rejected tumor [85]. The presence of TRMs has also been found to correspond with melanoma control as impaired TRM formation has been associated with an increased susceptibility to developing uncontrollable cancer and metastases [99]. Furthermore, the tissue retention markers VLA-1 and CD103 are important for melanoma tumor control as in vivo blockade weakens control of subcutaneously engrafted melanoma tumors [100]. However, independently of CD103 expression, CD8^+^ T cells in melanoma tumors have a TRM gene signature. Additionally, not only CD8^+^ but also CD4^+^ lymphocytes have been implicated in tumor control [61].

The expansion of tumor-resident CD8^+^, CD103^+^ T cells have been shown to predict treatment response in patients treated with immune checkpoint inhibitors [119]. Furthermore, a case report corroborated that TRMs were involved in melanoma cancer-immune surveillance and cancer-immune equilibrium [31]. CD8^+^ CD103^+^ TRM cells bearing the marker CD39, which is involved in immunosuppression, have been found in increased numbers in melanomas compared with a variety of other tumor types [32]. Higher frequencies of CD8^+^ CD103^+^ lymphocytes bearing the CD39 marker were associated with better overall survival in head and neck cancers [32]. CD8^+^, CD49^+^ and CD103^+^ TRMs have been shown to be increased in perilesional melanoma skin during nivolumab treatment, potentially mediating protection against melanoma [101]. Runx3 has also been shown to be a key driver in lymphocyte accumulation and differentiation in melanoma, potentially pointing to a future treatment target [102]. An understanding of the characteristics of TRMs in melanomas and that factors regulating them will allow investigation of new treatment options for patients with melanoma.

### 3.5. Cutaneous T-Cell Lymphoma

CTCL encompasses both leukemic forms of the disease, including Sézary syndrome and fixed skin-limited variants such as mycosis fungoides (MF). T cells from MF skin lesions have a nonmotile nature and a memory cell phenotype, and can therefore remain fixed in skin locations for a long time [26]. Multiple eruptions of adult T-cell lymphoma might be due to migration of skin-infiltrating pathogenic TRMs [33]. Furthermore, a case study on a patient with CD8^+^ primary cutaneous peripheral T-cell lymphoma found an infiltration of T cells with a TRM phenotype [34]. Alemtuzumab, an anti-CD52 antibody targeting mature lymphocytes of both T and B cell origin, has been shown to deplete all circulating and recirculating T cells including central and effector memory T cells, but sparing TRMs, thus effectively treating leukemic CTCL but not MF [62,63]. Using alemtuzumab as a model for T cell depletion, the same study found that almost all CD4^+^ and CD8^+^ T cells in CTCL skin from human patients predominantly expressed CD69, whereas CD103 was primarily expressed on CD8^+^ T cells in the epidermis. CD103^+^ TRMs had a higher production of IFN-γ, TNFα and IL-22 than CD103^−^ TRMs [63]. Epidermotropism of T cells is a histological hallmark in human CTCL. Epidermotropic lesions were found to harbor cells with a CD4^+^ TRM phenotype in a mice model of CTCL. Here, TRMs accumulated around hair follicles. The authors also showed that IL-15 and IL-7 might inhibit the epidermotropism, and therefore serve as important future targets for inhibiting TRM accumulation in the skin in CTCL [35].

### 3.6. Others

TRMs may play a role in many other fixed and non-fixed skin diseases (Table 2). In our systematic literature search, we identified studies showing that CD69^+^/CD103^+^ TRMs were increased in lesional skin from the scalp in patients with frontal fibrosing alopecia and alopecia areata, compared with non-lesional and normal scalp skin [115]. In chronic graft-versus-host disease, autoreactive CD4^+^ TRMs recognize autoantigens presented by B cells to enhance production of IgG autoantibodies that augment skin damage [64]. Another study on donor cells from facial allograft rejection cases expressed increased CD69, CD103 and conjugated linoleic acid (CLA) biomarkers [36]. TRMs were also involved in toxic epidermal necrolysis [66], drug eruption following herbal medicine [37], systemic sclerosis [39] and cutaneous drug hypersensitivity reactions [40]. The skin of patients with drug hypersensitivity reaction associated with alemtuzumab treatment was also found to harbor CD8^+^ CD103^+^ TRMs [104].

TRMs have been shown to be expanded in atopic dermatitis lesions [67,68], with half of TRMs in dermis co-expressing the immune checkpoint inhibitor-programmed cell death protein 1 (PD-1). Enhanced PD-1 expression might characterize memory cells with enhanced effector function, thus positioning PD-1 inhibition as a future target [68].

Using 1-Fluoro-2,4-dinitrobenzene applied to mice as a model of allergic contact dermatitis, another study established that local disease memory is associated with the accumulation in the epidermis of CD8^+^ TRMs that are able to produce IL-17A and IFN-γ [69]. Using the same model, other groups corroborated the accumulation of epidermal CD8^+^CD69^+^CD103^+^ TRMs in affected skin [41,70,105]. The magnitude of this allergic reaction correlated with the accumulation of CD8^+^ epidermal TRMs, which in turn correlated with allergen dose and number of allergen exposures [41].

Healed chronic hypersensitivity lesions are also enriched for allergen-specific CD8^+^ TRMs, which persisted in the skin for months [70]. Healed contact hypersensitivity skin of BALB/c mice also contained increased numbers of CD4^+^ and CD8^+^ TRMs, which were responsible for disease flare-up [42]. Using a contact sensitizer hapten, a protein, and a virus, a generation of TRMs in the skin after repetitive antigenic challenges could be observed [72]. Dendritic cells are also able to prime the creation of TRMs in inflamed murine skin [106]. Another study using a contact protein showed that TRMs as well as their local increase following antigen exposure are important for sufficient control of hypersensitivity responses [43]. Furthermore, long-lasting immune protection against locally encountered antigens depends on the induction of CCR8^+^ TRMs [44].

In actinic keratosis, a short course of topical calcipotriol plus 5-fluorouracil treatment on the face and scalp has been shown to induce immunological memory in the skin by contributing to TRM formation [45].

In summary, it is possible that CD8^+^ TRMs are responsible for the development of flares in eczema, allergic contact dermatitis, and fixed drug reactions, as well as for immunological memory, causing protection against ulcers in systemic sclerosis, protection against actinic keratoses, and protection against rejection in graft-versus-host disease. It has also been shown that CD8^+^ TRMs can serve as a driver of disease in frontal fibrosing alopecia and alopecia areata.

## 4. Discussion

The aim of this study was to conduct a systematic review of current literature on the role of TRMs in skin diseases. Knowledge of skin TRMs is rapidly evolving, and it appears that pathogenic TRMs are present in many chronic skin diseases. However, even though TRM presence is dictated by the local microenvironment and cytokine milieu, many critical questions remain. It thus remains unclear whether TRMs contribute to pathology in an indirect or direct manner.

Research in both mice and humans has contributed to determine the phenotype and function of TRMs in skin diseases. However, techniques are lacking for detecting TRMs in tissues with higher accuracy, limiting knowledge of their generation, survival and transit. One major caveat is that TRMs are only putatively stable in the skin, whereas the pathogenic role of TRMs is amplified by their ability to remain in specific niches over the long term. Overall, surface expression was found to delineate TRM specialization in human skin, and correlates with the effector cell balance found in distinct inflammatory skin diseases. The included articles encompassed a heterogeneous definition of TRMs based on both surface markers and localization of cells in the skin. Additional work is needed to identify additional surface markers that might better define the pathogenic subset of T cells and tissue residency and determine the stability of the different T-cell subsets in the skin.

The studies highlighted in this review have focused on CD8^+^ TRMs, whereas the role of CD4^+^ TRMs in skin remains more unclear. In the lung, CD4^+^ TRMs contributes to a delayed infection [124], providing immunity against viral infection [125], and is necessary for CD8^+^ TRM generation [126]. However CD4^+^ TRMs may also cause lung fibrosis [127] and Crohn’s Disease [128]. The ability of CD4^+^ TRMs to be a cause of skin diseases both directly and through the effect on CD8^+^ TRM generation is thus very plausible in skin diseases. Future studies should therefore include local helper TRMs in the panel to further elucidate their function in skin diseases.

We acknowledge several limitations of the present review. Firstly, articles on TRMs from other peripheral tissues were beyond the scope of t this review. TRMs share similarities across tissues and therefore treatments that work on, for example, mucosal TRMs, may also influence TRMs from the skin. Secondly, not many studies have been conducted directly on human skin TRMs. Most studies have used mouse models of various skin diseases. These models are known to have inherent limitations in regards to the translatability of trafficking of lymphocytes [129].

The long-lived nature of TRMs may be a challenge in some diseases (e.g., psoriasis). Whether early therapy can change or modulate TRMs remains an interesting hypothesis that is currently being investigated [118]. Furthermore, it remains to be investigated to what extent changes in the microenvironment can influence the creation of long-lived pathogenic TRMs in skin. Studies on the composition of the skin microbiome and its impact on the training of skin TRMs are warranted. Furthermore, studies investigating how widely used dermatologic treatments (e.g., topical corticosteroids, phototherapy, small molecules and biologics) affect the various TRMs are still critically needed. Given the involvement of TRMs in autoimmunity and cancer, future research will hopefully focus on ways to block signaling pathways that inhibit effector function or induce apoptosis in pathogenic TRMs. In future, topical vaccination strategies for various skin diseases may also be a possibility.

## 5. Conclusions

In conclusion, this systematic review highlighted the critical role of TRMs in skin diseases and the enormous potential for harnessing skin TRMs for vaccines or immunotherapies. TRMs may be seen as potential key treatment targets, and targeting TRMs may be the key to disrupting the chronic course of many skin diseases.

## Figures and Tables

**Table 1:** Tissue-resident memory T (TRM) cell markers used by authors to define skin TRM cells. Often, a combination of markers was used. For the most part, TRM cells were either defined as CD3^+^, CD4^+^ or CD8^+^ T cells with an expression or absence of different markers. CD49a (α-subunit of the α1β1 integrin receptor) is another marker with important delineating functional implications. CD69 suppresses sphingosine-1-phosphate receptor 1 expression, which prevents T cell egress from tissues into the circulation. CD103 binds E-cadherin on epithelial cells, which mediates retention of T cells in the skin.

TRM Marker	Study
CD3	[18,27,28,29,30,31,32,33,34,35,36,37,38,39,40,41,42,43,44,45,46,47,48,49,50,51,52]
CD4	[18,26,27,31,33,34,35,36,37,39,40,41,42,44,45,48,49,50,51,52,53,54,55,56,57,58,59,60,61,62,63,64,65,66,67,68,69,70,71,72,73,74,75,76,77]
CD8	[14,18,27,28,29,30,31,32,33,34,35,36,37,38,40,41,42,43,44,45,46,47,48,49,51,52,53,54,55,56,57,58,59,60,62,63,65,66,67,68,69,70,71,72,73,74,75,76,77,78,79,80,81,82,83,84,85,86,87,88,89,90,91,92,93,94,95,96,97,98,99,100,101,102,103,104,105,106,107,108,109,110]
CD27	[34,62]
CD39	[32]
CD44	[34,35,43,50,64,65,76,92]
CD45RA	[34,52]
CD45RO	[26,37,40,52,62,66,73,74,111]
CD49a	[27,51,80,84,101]
CD62L	[34,35,50,52,64,65,71,72,76,83,84,92]
CD69	[14,18,27,28,29,30,31,32,33,34,35,36,38,39,40,41,42,44,45,47,48,49,50,51,52,53,54,55,57,59,60,61,63,64,66,67,68,69,70,71,72,73,74,75,76,77,80,81,82,83,85,87,88,89,90,92,93,95,98,99,100,105,106,108,112,113,114,115]
CD103	[14,18,27,28,29,30,32,33,34,35,36,38,39,40,41,42,43,44,45,47,48,51,52,53,54,57,58,59,60,63,64,65,66,67,69,70,71,72,73,74,75,76,77,78,79,80,81,82,83,84,85,86,87,88,89,90,92,93,94,95,96,98,99,100,101,102,103,104,105,106,108,109,112,113,115,116]
CD122	[71,72,76]
CD127	[76]
CCR4	[34,62]
CCR6	[52,54]
CCR7	[34,52,55,61,73]
CCR8	[44]
CLA	[36,54,59,62]
CXCR6	[64]
KLRG1	[84]
TCRα/β	[34]
TCRγ/δ	[34]
VLA-1	[100]
Vγ3	[41]
Vγ4	[117]
Vδ4	[117]
γδ	[117]

**Table 2:** List of diseases. Tissue-resident memory T cells were mostly investigated in various infectious skin diseases.

Disease	Study
Skin infections	
Viral skin infection	[14,38,46,47,48,53,54,75,76,77,82,83,84,85,86,87,88,90,91,92,105]
Herpes simplex virus-1	[18,65,71,78,79,80,89,93,97,103,107,108,109,110]
Candida albicans	[49,54,112]
Tick infection	[50]
Staphylococcus aureus	[113]
Herpes zoster	[74,81]
Leishmaniasis	[55,56]

Psoriasis	[27,51,52,57,58,59,94,114,116,117,118]
Melanoma	[30,31,32,60,61,73,85,96,98,99,100,101,102,119]
Cutaneous T-cell lymphoma or mycosis fungoides	[26,33,34,35,62,63]
Vitiligo	[27,28,29,57,95,120]
Others	
Acute localized exanthematous pustulosis	[37]
Drug reaction with eosinophilia and systemic symptoms	[40]
Skin allograft rejection	[36]
Chronic antigen exposure	[44]
Contact hypersensitivity	[42,43,70]
Skin contact hypersensitivity	[72]
Dinitrofluorobenzene-induced skin inflammation	[105,106]
Frontal fifibrosing alopecia	[115]
Drug hypersensitivity reaction	[104]
Contact dermatitis	[41]
Atopic dermatitis	[67,68]
Contact allergy	[69]
Actinic keratosis	[45]
Systemic sclerosis	[39]
Graft-versus-host disease	[64]
Toxic epidermal necrolysis	[66]

## Data Availability

Data supporting the conclusion of this study can be found in the supplementary tables.

## Supplementary Materials

The following are available online at https://www.mdpi.com/article/10.3390/ijms22169004/s1.
