# Supplementary material for: Tissue-Resident Memory T Cells in Skin Diseases: A Systematic Review
Source: Int J Mol Sci. 2021 Aug 20;22(16):9004. doi: 10.3390/ijms22169004 (PMC8396505; doi:10.3390/ijms22169004)
Supplement: Supplementary file 1 [file ijms-22-09004-s001.zip › PRISMA-S Checklist.pdf]

PRISMA-S Checklist

Section/topic	#	Checklist item	Location(s) Reported
**INFORMATION SOURCES AND METHODS**			
Database name	1	Name each individual database searched, stating the platform for each.	In title
Multi-database searching	2	If databases were searched simultaneously on a single platform, state the name of the platform, listing all of the databases searched.	Not applicable
Study registries	3	List any study registries searched.	Listed under methods
Online resources and browsing	4	Describe any online or print source purposefully searched or browsed (e.g., tables of contents, print conference proceedings, web sites), and how this was done.	Not applicable
Citation searching	5	Indicate whether cited references or citing references were examined, and describe any methods used for locating cited/citing references (e.g., browsing reference lists, using a citation index, setting up email alerts for references citing included studies).	Listed under methods
Contacts	6	Indicate whether additional studies or data were sought by contacting authors, experts, manufacturers, or others.	No additional studies were sought using listed sources
Other methods	7	Describe any additional information sources or search methods used.	Listed under methods
**SEARCH STRATEGIES**			
Full search strategies	8	Include the search strategies for each database and information source, copied and pasted exactly as run.	Listed under table S2
Limits and restrictions	9	Specify that no limits were used, or describe any limits or restrictions applied to a search (e.g., date or time period, language, study design) and provide justification for their use.	Listed under methods

Search filters	10	Indicate whether published search filters were used (as originally designed or modified), and if so, cite the filter(s) used.	No search filters were used on the databases
Prior work	11	Indicate when search strategies from other literature reviews were adapted or reused for a substantive part or all of the search, citing the previous review(s).	No search strategy from other literature studies were included
Updates	12	Report the methods used to update the search(es) (e.g., rerunning searches, email alerts).	No rerunning was performed
Dates of searches	13	For each search strategy, provide the date when the last search occurred.	Listed under methods
**PEER REVIEW**			
Peer review	14	Describe any search peer review process.	Listed under methods
**MANAGING RECORDS**			
Total Records	15	Document the total number of records identified from each database and other information sources.	Listed under figure 1
Deduplication	16	Describe the processes and any software used to deduplicate records from multiple database searches and other information sources.	Listed under methods

PRISMA-S: An Extension to the PRISMA Statement for Reporting Literature Searches in Systematic Reviews
Rethlefsen ML, Kirtley S, Waffenschmidt S, Ayala AP, Moher D, Page MJ, Koffel JB, PRISMA-S Group.
Last updated February 27, 2020.
