# Supplementary material for: Tissue-Resident Memory T Cells in Skin Diseases: A Systematic Review
Source: Int J Mol Sci. 2021 Aug 20;22(16):9004. doi: 10.3390/ijms22169004 (PMC8396505; doi:10.3390/ijms22169004)
Supplement: Supplementary file 1 [file ijms-22-09004-s001.zip › tableS2_2021june30.pdf]

Table S1. Search strategy used for the different databases.

**EMBASE:**
#1: "resident memory*" NEAR/2 (t OR cell OR cells OR lymphocytes)
#2: "skin-resident memory*" NEAR/2 (t OR cell OR cells)
#3: "tissue memory*" NEAR/2 (t OR cell OR cells)
#4: #1 OR #2 OR #3
**PUBMED:**
"skin-resident memory*" OR
"resident memory" OR
"resident memory t*" OR
"t(rm)" OR
"tissue memory" OR
"tissue resident memory*" OR
"tissue resident memory cell" OR
"tissue resident memory t cell"
**SCOPUS:**
"resident memory*" W/2 (t OR cell OR cells OR lymphocytes), OR
"skin-resident memory*" W/2 (t OR cell OR cells), OR
"tissue memory*" W/2 (t OR cell OR cells), OR
**Web of Science:**
"resident memory*" NEAR/2 (t OR cell OR cells OR lymphocytes), OR
"skin-resident memory*" NEAR/2 (t OR cell OR cells), OR
"tissue memory*" NEAR/2 (t OR cell OR cells), OR
